# Flipped Learning 4.0. An extended flipped classroom model with Education 4.0 and organisational learning processes

**DOI:** 10.1007/s10209-022-00945-0

**Published:** 2022-11-19

**Authors:** María Luisa Sein-Echaluce, Ángel Fidalgo-Blanco, Ana María Balbín, Francisco José García-Peñalvo

**Affiliations:** 1 Department of Applied Mathematics, EINA, Universidad de Zaragoza, Calle de María de Luna 3, 50018 Saragossa, Spain; 2 Laboratory of Innovation in Information Technologies. LITI, Universidad Politécnica de Madrid, Calle de Ríos Rosas 21, 28003 Madrid, Spain; 3 Education Faculty, Pontificia Universidad Católica del Perú, Av. Universitaria 1801, San Miguel, 15088 Lima Perú; 4 Department of Computer Science and Automation, Science Faculty, Universidad de Salamanca, Plaza de los Caídos S/N, 37008 Salamanca, Spain

**Keywords:** Education 4.0, Flipped classroom, Knowledge creation, Organisational learning, Cooperative learning, COVID-19

## Abstract

This article integrates two visions on the creation of knowledge by students: an academic vision where the person who creates knowledge uses high-level cognitive abilities and, therefore, acquires deeper learning, and an organisational learning vision, where the creation of knowledge adds value to the organisation and the individuals who work in this matter. It starts from a validated flipped classroom model and then adds procedures and cycles of knowledge that make it an active methodology, in such a way that it simultaneously supports organisational learning, using cooperative competencies characteristic of Education 4.0. This proposed hybrid model has been applied online during confinement due to the COVID-19 pandemic and, subsequently, in dual mode (students partly in person and the rest online at the same time) and face-to-face mode. The evidence of this research shows that the creation of knowledge by the students, cooperatively and with an organisational learning perspective, has repercussions for improvements in their academic performance by producing deeper learning. In addition, the development of cooperative skills is observed to create and manage a large amount of helpful knowledge for them and other students in their learning process.

## Introduction

The flipped classroom (FC) method places the emphasis on reversing the learning process. More specifically, it reverses the order in which two of the most common activities in the training process occur: the “lesson” and the “homework”. Whereas in a traditional and common model, the “lesson” is done in class and the “homework” is done at home, in the FC methodology, the “lesson” is done at home and the “homework” is done in class [1].

From an academic perspective, the accomplishment of homework includes cognitive activities of a higher level than those performed by listening to only one lesson, especially if these activities are carried out cooperatively and with the advice and supervision of teachers. This idea is what makes the FC methodology active [2, 3].

From the academic point of view, regarding the impact of the method itself, the students positively value the active methodology [4–8]. It can be used in any academic setting [9] and optimises the time spent learning [10, 11].

Regarding learning, the academic results are improved in traditional exams (summative assessment) by using higher-level cognitive abilities [4, 12, 13], the acquisition of teamwork competence [5], and practical classes involving problems, laboratory assignments and projects [14, 15]. It also reduces the students’ perception of the complexity of the course content [16], improves students’ level of achievement in the course [17, 18], and increases the self-efficacy of learning [19, 20] and the adaptation to the course at the student’s own pace [11]. Likewise, the FC method increases students’ level of motivation [21, 22] and sense of individual responsibility for their own learning [23] and collective responsibility when working on a team [6].

From the perspective of cooperation, the FC method favours peer learning [6, 24] and increases discussions [6], interactions between students [11, 25] and student participation in activities [21].

From the perspective of content creation, the FC method allows students to create content [4], which can be used as learning resources by other students. Thus, the FC method transforms the role of the student into a producer–consumer of content [26, 27]. This enables increasing the knowledge provided in the course itself from the students’ knowledge and experience, producing organisational learning [16, 28]. The students are also capable of organising the knowledge created individually and collectively [24, 29], and in all of this, the students apply high cognitive abilities [10].

In addition, because of the restrictions on access to classrooms during the confinement due to the COVID-19 pandemic [30, 31] and the subsequent hybrid teaching models [32, 33], when limitations of capacity and social distance had to be respected, the FC method has been one of the primary references for many teachers [34–36], especially for teachers who wanted to maintain an active learning method and not just call hybrid learning the mere retransmission of the face-to-face class session by videoconference for those students who could not physically attend the classroom.

Through bibliographic reviews, including the authors’ experience in applying the FC method for more than 9 years [37], FC is shown to be an active method that results in improvements in the learning results in theoretical and practical classes and the acquisition of teamwork competence, increasing the students’ responsibility in their own learning process, as well as increasing interactions and debates among the students themselves.

However, in Education 4.0 [38, 39], adapted to new industrial and competitive needs, emphasis is placed on the enhancement of cooperative capacities, on the creation of open knowledge in a cooperative way and on the management of all this knowledge. On the other hand, organisational learning goes a step beyond the creation of knowledge, incorporating its management and use to favour the learning of individuals but also of the organisation.

Likewise, the bibliographic review has shown that the FC method favours cooperation between individuals, the creation of knowledge and students’ use of knowledge.

The purpose of this work is to integrate characteristic processes of organisational learning and Education 4.0 with the processes of the FC method. In this way, cooperation between students is increased, as well as the creation of open knowledge, its organisation together with the acquired experience and its use as a learning resource in the subject.

The main objective of this work is to define and apply an FC method that supports organisational learning in a course using the management of knowledge and the experience acquired by students during the completion of the course. Likewise, to accommodate the 4.0 model, the knowledge created in the context of the subject will be open and accessible both to students and to the rest of society.

The objectives of this work are as follows:

1. To define an FC model that can support organisational learning incorporating Education 4.0 competence for cooperation in the creation and management of open content;
2. To apply this model to obtain evidence that the developed model maintains the impact on active learning, like any other FC method, as well as the 4.0 competencies in terms of cooperation and organisational learning.

The following sections detail the FC model applied in this research, followed by the results of the case study of the research carried out, ending with the discussion and conclusions of the work.

## Functional model

The objective of this section is to identify the processes that are incorporated into the FC methodology to support organisational learning and 4.0. The traditional processes of the FC method are aimed at enhancing students’ active learning, originally in theory classes and later in other learning scenarios such as practical classes and during the acquisition of horizontal competencies, such as teamwork. The incorporation of cooperative processes for the creation of knowledge, as well as processes to manage and use the knowledge created, generates the model that we call Flipped Learning 4.0 in this work.

**Fig. 1:**
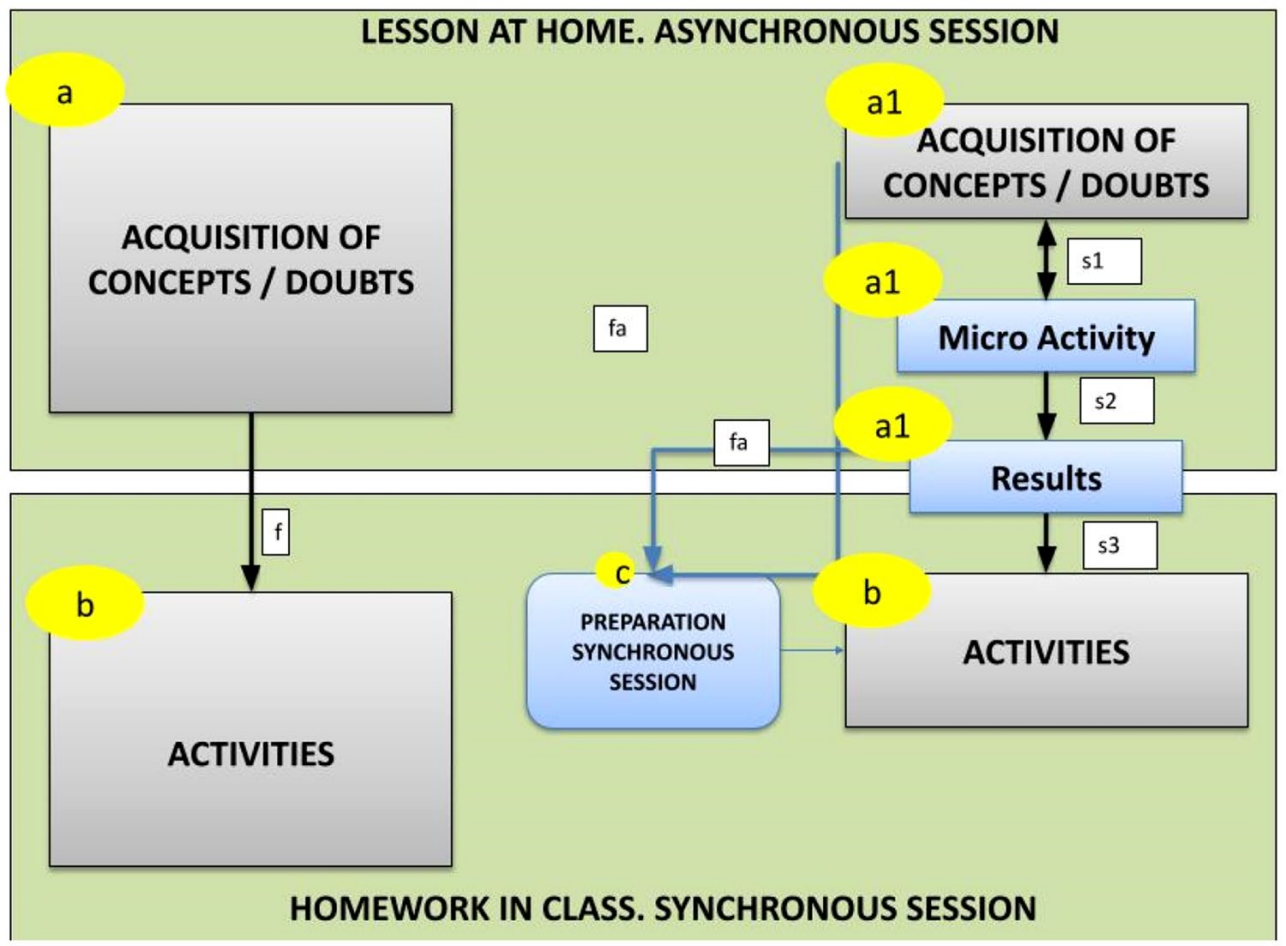
Comparison of classic FC models with the MFT model

Figure 1 (a and b) shows the processes of the initial FC model [40, 41]. The model is made up of two processes: the lesson at home and the homework in class [1]. The home lesson is composed of a process whose mission is for students to learn the lesson through the acquisition of certain concepts (Fig. 1a) and which is usually completed through communication processes with students so that they can raise questions and comments. The flow “f” that connects the home lesson with the homework in class is based on the knowledge that students have acquired during the home lesson.

The homework in class (Fig. 1b) is usually worked on from questions students raised about the concepts acquired in the lesson at home, debates are promoted, and practical activities are carried out. This model assumes that students bring the lesson learned during the asynchronous session to the synchronous session. During the home lesson, the students tend to have a passive attitude, whereas during the homework in class, their attitude is usually more active.

The authors of this research developed an FC model called MicroFlipTeaching (MFT) [4, 5] that substantially changes the processes of the lesson at home and the homework in class with respect to the traditional model. During the lesson at home, it is intended that the students also have an active attitude, and for this, instead of the teacher taking charge of the lesson (as in the classical model, where teachers describe the concepts of the lesson), they carry out a practical micro-activity from the acquisition of concepts. The idea is to work with a part of the lesson rather than the entire lesson.

Thus, this MFT model (Fig. 1, a1) includes three processes: the acquisition of concepts (similar to the classical model but working only with the concepts necessary to carry out the micro-activity), the micro-activity (a practical application that can be carried out individually or cooperatively) and the generation of results (from the micro-activity). All of this, as in the classic model, is complemented with communication with the students to raise questions and queries.

In the home lesson of the MFT model, the flows are as follows:

- “S1” represents the knowledge acquired to complete the activity (this flow has two senses; first, they can try to complete the micro-activity and then acquire the concepts, and vice versa);
- “S2” corresponds to obtaining the results of the micro-activity.

In the MFT model, a new process is generated (Fig. 1b) that is based on the capture of evidence of the interaction (flows “fa”) of the students with the processes of the lesson at home. With this evidence, teachers can decide what resources to prepare during the synchronous session corresponding to homework in class. These data can be observed manually by teachers, such as seeing the results of the micro-activity, the doubts raised or the interaction with the resources where the concepts are exposed. They can also be analysed by learning analytics systems [42, 43] through, for example, the interaction data provided by the e-learning platform (resources viewed, dates, duration, messages in forums, etc.).

The processes corresponding to the homework in class also change because initially one works with the results of the micro-activities (flow “s3”). One works with both wrong and correct results. It is in this analysis of results where debate, reflections and cooperation are promoted to correct or validate the results reported by the students and that have been analysed.

Thus, in the MFT model, the home lesson objective is not for the students to take the lesson learned to the synchronous session, but rather for them to carry out the micro-activity, whether the results are wrong or correct. Likewise, in the homework in class, the idea is to give micro-lectures to complete the lesson’s contents and practical and participatory activities.

On the other hand, both the classical FC model [2, 3] and the MFT model [4, 6, 44] have been shown to be active methodologies. In the case of the MFT model applied to work teams, it has been shown that the cooperative process is transparent [45] for both the team and the entire teaching group, that there is shared leadership [43] based on values [42] and that teamwork skills [6] and cooperation are acquired for the creation of knowledge [5]. The 4.0 learning model requires cooperative skills [38, 46], and in this sense, the MFT method already includes them.

In this work, the incorporation of new processes into the MFT method is provided to support organisational learning and the competencies of Education 4.0 in terms of content creation (because cooperative skills already use the MFT model).

**Fig. 2:**
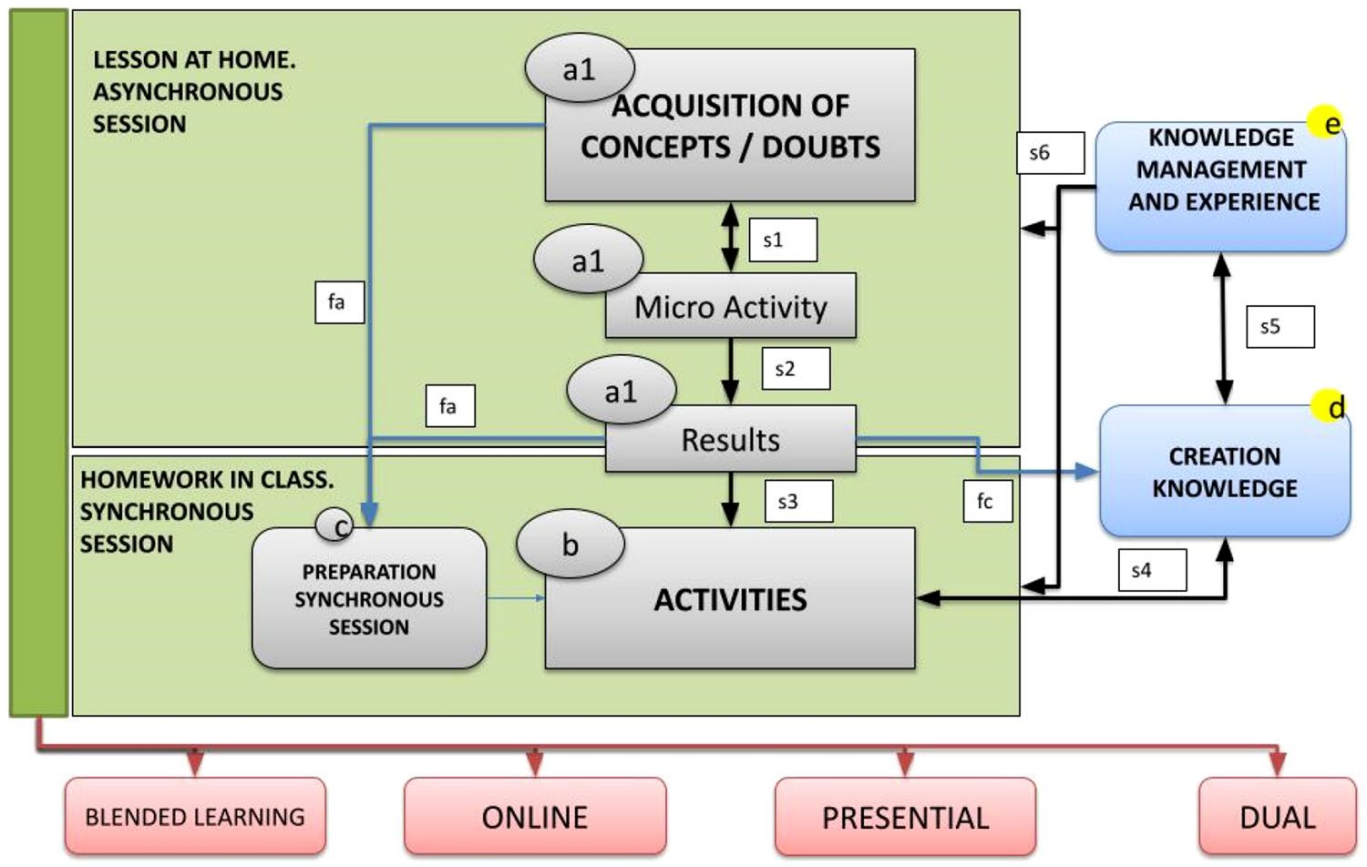
Flipped Learning 4.0: The MFT model with the processes for organisational learning

Figure 2 shows the new processes and flows incorporated into the MFT method to adapt them to the organisational learning model. This incorporation is what gives rise to the Flipped Learning 4.0 model. The main objective of incorporating these new processes is to support the creation of open knowledge by students, as well as the management of said knowledge and the experience of its creation.

The main processes by which students create knowledge are the results of the micro-activities (process belonging to the lesson at home) and the homework in class, where they work with the results of the micro-activities and practical activities. Knowledge is usually obtained and refined at two levels:

- Level 1—During the results of the micro-activity. At this level, the knowledge can be correct or incorrect. For this reason, a second level of refinement is needed;
- Level 2—Level 1 knowledge is refined. If level 1 knowledge is wrong, errors are identified and corrected. If level 1 knowledge is correct, it can be improved by incorporating reinforcements, for example, structuring it in a way that makes the disclosure easier, including comments or incorporating other clarifying elements.

The creation of knowledge through the two levels is reflected in the process “e” of Fig. 2. The knowledge of the first level is represented by the flow “fc” and that of the second level by the flow “s4”. The double direction of the flow “s4” represents the possibility of changing the knowledge of the first level. The knowledge created in this process requires peer quality control; that is, the knowledge is reviewed by other students and ultimately by teachers.

The second incorporated process (Fig. 2e) is a knowledge management system where the students’ experience can also be incorporated for the creation of knowledge. This could include what part of the work has been more difficult, the time taken to create it, common mistakes, recommendations for the use of knowledge and more. Flow “s5” represents the incorporation of the created and validated knowledge into the knowledge management system.

In the knowledge and experience management system, resources are classified by types (problem, example, notes, survey, map, etc.), learning activity (conceptual and practical), the profile of the recipient (student who has not attended class, student who has attended class but has not understood the concept, etc.), subject and academic year. From the labels used for their classification, logical expressions can be built to facilitate their search.

Once the knowledge management system is available, it can be used as an additional resource to understand the concept and carry out the micro-activity of the home lesson. It can also be used by teachers to carry out activities of the homework in class processes and even by students to prepare for the subject exams. All this reuse is reflected in the flow “s6”.

The flows from “s1” to “s6” represent a spiral (cycle) of knowledge creation. This open knowledge, created by the students themselves, is useful for different purposes:

- For students while taking the course (both to carry out learning activities and to carry out assessment tests) and for teachers who can use it as a learning resource within the homework in class phase;
- For the same subject in later academic courses, so that the teachers prepare a micro-lesson of the homework in class to help in the acquisition of concepts of the lesson at home and so that the students of later courses receive help in academic learning and the creation of new knowledge.

Therefore, there is also a knowledge utilisation cycle.

The combination of the cycles of creation and use of knowledge is the basic principle of organisational learning [47, 48]. In this organisational learning model, it is contemplated that there are inexperienced people who progressively learn until they are experts [49] (in this case, the enrolled students who had no experience in the subject acquire it and transmit this learning process to the organisation). The knowledge produced is useful for the people in the organisation and is created by a community of practice [50] (in the case of the subject, it is useful to carry out the learning activities of the students, and the community of practice is the students of the subject) and creates value for the organisation itself [51] (in this case, the organisation is the subject). Thus, this model is associated with the characteristic processes of organisational learning and incorporating the necessary competencies for cooperation in the creation, management and use of knowledge; it is also associated with the competences of Education 4.0.

Likewise, as represented in Fig. 2, the model is based on synchronism (temporal coincidence of teachers and students) and asynchronism (no temporal coincidence). In a fully face-to-face context, synchronism is the coincidence in the classroom and asynchronism outside the classroom. In a fully online context, both synchronism and asynchronism can be carried out with online technologies, as is the case in b-learning contexts. In the case of dual training (a percentage of the students are in the classroom and the rest are online), synchronism can be achieved when all the students (in the classroom and online) are using the same online technologies, so that the students who are in the classroom can cooperate with those who are online. For this reason, the method is hybrid and adapts to any learning situation, such as those originated by the COVID-19 pandemic [52–55].

## Context

To measure the impact of the model on academic learning, it is necessary to have a control group and an experimental group. However, the control group should not access the content generated by the students in the experimental group for effective comparison of results. On the other hand, to measure organisational learning and 4.0, it is necessary for the student-created content to be available in the open, managed once created and accessible (and usable) by the entire learning community. Thus, experimental and control groups cannot be established in this case, but the evidence of the learning community created can be analysed.

For this reason, this research has been carried out in two contexts: one to measure the impact of the model on academic learning (restricting access to content) and another to measure the contribution of organisational learning and 4.0 (where open content must be accessible online to the entire learning community).

The exposed model must achieve improvements in academic results, as with other FC models, but it must also support the development of learning using organisational skills from Education 4.0. Therefore, the model is analysed under these two approaches, and each of them is tested in an academic subject of different grades.

The verification of the improvement of academic results is carried out in the subject of “Computer Science and Programming” of the Degree in Mining and Energy Engineering (Context 1), whereas the support for organisational learning is analysed in the subject “Fundamentals of Programming” of the Degree in Biotechnology (Context 2). Both are official degrees from the Technical University of Madrid, and both subjects are taught in the first semester of the corresponding degree program. The sample was taken during the 2021–2022 academic year.

*Context 1—*The study on the improvement of academic results was carried out in the programming laboratories of the subject “Computer Science and Programming”. In this subject, there are three official academic groups with a total of 236 students, two groups in the morning and one in the afternoon.

Each academic group is divided into two subgroups for the programming laboratory, for which there are six laboratory subgroups (two subgroups with 50 people and four subgroups with 34 people). All laboratory subgroups work with the same materials, and the final exam has the same difficulty level for all.

Quasi-experimental studies have been carried out, involving two laboratory subgroups of 34 people each (one is the control group, and the other is the experimental group). These two laboratory subgroups have the same faculty, and the final exam was prepared by faculty not involved in this research.

*Context 2—*To investigate evidence that allows us to affirm that the model supports organisational learning and 4.0, we have worked with all the students of the subject “Fundamentals of Programming”. In total, there were 78 students divided into two groups, one in the morning and one in the afternoon. On the other hand, the subject consists of classes taught in the classroom and laboratories, and the research has been applied in all learning activities of the subject.

The following section presents the results related to both contexts.

## Results

The results for each of the scenarios of this research are presented below:

- Context 1—Impact of the model on academic learning outcomes;
- Context 2—Impact of the model on organisational learning support and 4.0.

### Context 1—Impact on academic learning outcomes

The control group (CG) and the experimental group (EG) correspond with two laboratory subgroups, with 34 people enrolled in each. Nine laboratory sessions were carried out, and during the first four sessions, both groups followed the same method. The experimental group followed the method based on the proposed model from the fifth to the ninth session. Class attendance was accounted for in those two periods.

Next, the results that support the homogeneity of the two groups considered—control and experimental—are shown for the characteristics of the students and in terms of the students’ perceptions regarding the treatment received by the subject teachers.

#### Homogeneity of the sample regarding the students of the two groups

Prior to the research, a survey was conducted for the control and experimental groups. Regarding the number of students enrolled in the subject, 38% participated in the CG and 35% in the EG. Regarding the average attendance during the first period (before applying the innovation), participation in the survey was 64.20% in the CG and 50% in the EG.

Questions regarding age, university entrance qualification (UEQ), gender, and the number of times they had enrolled in the subject were included in the survey.

The responses represent a non-normal distribution, and to check for significant differences, the Wilcoxon p-value [56] is used for a pair of unpaired samples. The results are presented in Table 1. The characteristics of the sample are homogeneous except for the number of times the subject is repeated.

**Table 1:** Homogeneous samples regarding comparison

	Age	UEQ	Gender	Enrolment number
Wilcoxon nonparametric *p*-value	0.8334	0.4696	0.9414	0.00000325

#### Homogeneity regarding the treatment received by the teaching staff

To verify the homogeneity regarding the treatment received by the students of the control and experimental groups, the variables that make up the dimension “Attention of the teaching staff received by the students” of the MUSIC survey [57] have been used and validated to measure the motivation of the student body. This survey was conducted after the implementation of the proposed model was completed.

Eight people participated in the CG and 14 in the EG. Regarding those enrolled, the participation percentage is 23.53% for the CG and 41.17% for the EG. Regarding the average class attendance (period of the 5th to 9th session), the percentage is 78.58% in the CG and 66.66% in the EG.

The items included in the survey are as follows:

Q1. The professor is available to answer my questions related to laboratory activities;

Q2. The teacher is willing to help me when I need it;

Q3. The teacher cares about my performance in the course;

Q4. The teacher is respectful to me;

Q5. The teacher is friendly;

Q6. I think the teacher cares about how I feel.

The responses obtained in all the variables correspond to a non-normal distribution. Therefore, to see if there are significant differences, the Wilcoxon p-value of the nonparametric comparison of two unpaired samples is calculated. Table 2 shows the results of this comparison.

**Table 2:** Contrast variables for homogeneity in the treatment received by the teaching staff

	Q1	Q2	Q3	Q4	Q5	Q6
Wilcoxon p-value	0.9042	1	0.7597	0.2888	0.4586	0.6073

Table 2 indicates that there are no significant differences in the treatment given by the teaching staff in the control and experimental groups.

Likewise, in this survey, the data on age, university access grade (UEQ), gender and number of times enrolled in the subject were gathered again. Whether there were significant differences between the samples was analysed through the Wilcoxon p-value for nonparametric samples. The results are included in Table 3, and on this occasion, there were no significant differences in any variable.

**Table 3:** Contrast variables on the homogeneity of the sample

	Age	UEQ	Gender	Enrolment number
Wilcoxon nonparametric *p*-value	0.967	0.3741	0.7798	0.5087

The academic results of the students in the control and experimental groups are included in what follows.

#### Academic results in context 1 for the CG and EG

In addition to the final laboratory exam, “Computer Science and Programming” students can do up to four volunteer jobs. Column 7 of Table 4 includes the mean number of assignments delivered by students enrolled in the group. These works can raise their exam grade if they get 3.3 out of 10 on the exam. Furthermore, if this grade is surpassed, it can be averaged with other exams of the subject. For this reason, the exam scores reflected in Table 4 (columns 4, 5 and 6) distinguish the scores obtained in “failures without a minimum mark” (column 4), “failures with a minimum mark” (column 5) and passed (column 6).

**Table 4:** Academic results of the control and experimental groups

Group	Class attendance (average of all sessions)	Presented to the exam	Suspended with mark between 0–3.2	Suspended with mark between 3.3–4.9	Approved ≥ 5	Mean of works completed by student
CG	43.14%	52.94%	83.3%	16.7%	0%	1.38
EG	65.69%	64.70%	45.45%	36.37%	18.18%	2.12

**Table 5:** Distribution of resources by learning activity

Acquire concept	Apply concept
146	105

The first column of Table 4 represents the average attendance percentage considering attendance at the nine sessions. Column 3 represents the percentage of students who presented to the exam with respect to the percentage enrolled in each group.

### Context 2 Impact on organisational learning support and 4.0

In this scenario, the impact of the proposed method on organisational learning and Education 4.0 is determined by analysing evidence generated by the students themselves. There were 78 participating students (from the Degree in Biotechnology) organised into 13 working groups with an average of six students per group.

From the perspective of organisational learning, the evidence on the creation, organisation and use of the knowledge created by the students is analysed. From the perspective of Education 4.0, cooperation is analysed for the group to create, organise and use the knowledge created.

#### Knowledge creation

Regarding the creation of knowledge, the students have created resources that collect the knowledge and experience acquired during the learning of the subject. In this sense, 243 knowledge resources have been created, for an average of 3.11 resources per participant in the subject.

The knowledge has been structured in the fields indicated below (this process is the one carried out through the flows “s2”, “s3” and “s4” in Fig. 2):

- Resource title: to identify the activity and topic of learning;
- Short description: so that users have a brief summary of the resource;
- Justification: to collect the need for the resource in its use during the learning of the subject;
- Recommendation for use: advice on how to use it within the course;
- Quality control: provides guarantees of the veracity of the knowledge. Quality control can be completed by peers (reviewed by all team members) or by the subject teaching staff;
- Resource content: usually a link to a file with the resources. The types of resources are usually videos or graphic texts.

#### Knowledge organisation

Once the knowledge is created, it must be classified, stored and organised in a free online access knowledge management system [1, 26, 58]. The knowledge classification was carried out by the students themselves, and they established various classification categories: learning activity (Table 5), recipient profile (Table 6), type of resource (Tables 7 and 8) and subject (Table9).

**Table 6:** Distribution of resources by student profile

“Before attending class”	“Lost”	“All understood”
84	151	91

**Table 7:** Types of resources

T1	Notes
T2	Authorised notes for exam
T3	Questionnaire
T4	Doubts
T5	Example
T6	Exercises
T7	Polls
T8	Interviews
T9	Mistakes
T10	Exam
T11	Explanation
T12	Map
T13	Summary
T14	Tips
T15	Lesson learned

Each category is made up of a set of tags that enables classifying knowledge, organising it dynamically and searching for it [59].

Next, the tables corresponding to each category are analysed. The columns indicate the labels for each category as the number of knowledge resources associated with each category. It should be noted that the same resource can have several labels; therefore, the number of resources per element might not correspond with the total number of elements.

Table 5 shows the category “learning activity”, which expresses the type of activity to be carried out with the resource. The students divided this category between a conceptual aspect (acquiring the concept) and another practice (applying the concept).

Table 6 reflects the labels established for the “student profile” category. This category is closely related to the “recommendation for use” because the resource is meant to be used in a specific situation from the perspective of student class attendance.

The label “before going to class” indicates that it is a resource whose recommendation for use is before attending class or laboratory, for example, to get an idea of the content to be taught, the necessary prior knowledge, the complexity and so on.

The label “lost” represents that the resource is aimed at students who have attended class or laboratory but have not understood how to carry out a particular learning activity.

The “all understood” label usually includes resources for expanding knowledge or requiring a certain complexity. They are intended for students who attend learning activities and know how to complete them, so they want to learn more.

Table 7 lists the different types of resources that students created, and Table 8 lists the number of resources created for each type. The labels represent the different types of materials that are needed to carry out the learning activities. Some of them are common, such as summaries, examples and exams, and others are not as common, such as interviews, tips (tricks), lesson learned (explanations of how they have organised the cooperation) and authorised notes (material that some teachers authorise during the exam).

**Table 8:** Distribution by type of resource

T1	T2	T3	T4	T5	T6	T7	T8	T9	T10	T11	T12	T13	T14	T15
80	14	30	13	74	57	10	9	16	21	83	8	54	32	26

**Table 9:** Distribution by thematic blocks

Classroom Classes	Laboratories	Cooperation
125	67	36

The subject consists of three thematic blocks corresponding to different learning activities: classroom classes (numerical algorithms), laboratories (programming in R language) and cooperation (teamwork). Table 9 reflects the number of resources generated in these thematic blocks.

#### Use of resources and cooperative work

The resources included in the knowledge management system have been visited 12,947 times, with an average of 53.28 visits per resource.

Regarding interaction, forums have been used in the Moodle course of the subject for the organisation of each team. They have also used other means such as WhatsApp and videoconferencing systems. The evidence has only been collected from the Moodle forums because the teachers do not have access to WhatsApp or videoconferences. The number of messages exchanged in the forums is 6076, which corresponds to an average of 76 messages per student.

Regarding the number of documents on coordination that each team has used internally, 93 internal documents have been generated.

## Discussion

In previous studies carried out in different educational settings [9, 60, 61], indicators were identified that measure the active participation of students, such as class attendance, taking the exam and submitting work. In all the mentioned indicators, the experimental group obtained better results than the control group. These results confirm those obtained by other authors on the relationship between the FC method and the active participation of students both in previous situations to the circumstances of the restrictions implemented due to the COVID-19 pandemic [2, 3], as well as during those circumstances [62].

It should be noted that a variable (number of times enrolled in the subject) was included in the contrast of results at the beginning of the research, in which there were significant differences between the control and experimental groups. There were more repeaters in the EG than in the CG; however, this difference gradually disappeared during the research because the few students who stopped attending the laboratory sessions in the EG were students who had already studied the subject in previous courses.

From an academic point of view, the creation of knowledge, as well as the cooperation to create it, is considered a characteristic of active methodologies [63, 64], as well as the improvement of learning, because it uses high cognitive abilities [65, 66]. In this sense, the academic results reflect the increase of the students’ cognitive abilities to solve problems (laboratory test). It is observed that more than 54.5% of the EG students presented to the exam, passed or exceeded the minimum qualification necessary to make averages between exams and assignments, whereas in the CG, this percentage is 16.7%.

In the academic field, the creation of knowledge is associated with improving the cognitive abilities of the person who creates it. In the organisational vision, a dimension is added, which is the value that this created knowledge contributes to the rest of the people in the organisation and to the organisation itself [48]. On the other hand, Education 4.0 seeks to train students to create and manage knowledge [67, 68]. Therefore, it could be said that cooperative skills for the creation of knowledge provide support for organisational learning.

Concerning cooperation, the number of messages exchanged in the forums and the creation of resources that reflect the coordination and cooperation between teams to create knowledge demonstrate the high impact of applying these skills. This aspect coincides with other studies highlighting the increase in interactions among students using the inverted classroom method [69], as well as increased cooperation among the student body during the COVID-19 pandemic restrictions [70]. In addition, the students have structured, classified and use knowledge through a knowledge management system. All evidence of the application of the Education 4.0 competencies aligns with other investigations [38, 71, 72].

In organisational learning, emphasis is placed on the usefulness of the knowledge created for the activities of the organisation itself [50], as well as its organisation and management [73, 74]. The knowledge created by the students corresponds to the activities of the organisation, which in this case are learning activities because the organisation is a university course. Therefore, this content is beneficial not only for the students who created it but also for the subject itself because this knowledge is accessible to students in later courses and even for other subjects.

## Conclusions

An FC model has been designed that incorporates the skills of Education 4.0 regarding cooperation for the creation of knowledge and its management, following the principles of organisational learning. For this, processes have been incorporated to create and manage knowledge and knowledge spirals that enable its flow during the teaching of the subject so that it can be used in the learning activities that integrate them.

This new model continues to be valid for its application as an active methodology because the results of class attendance have validated it: the delivery of proposed works and the percentage of students who take the exam in the EG compared with the CG (Table 4).

One of the novelties that this new model incorporates is the students’ creation of open knowledge in a cooperative way. This creation requires high-level cognitive abilities, which ensure deeper learning on the students’ part. Laboratory tests requiring high cognitive abilities show that EG has significant differences from the CG (Table 4).

The use of the model implies competencies characteristic of Education 4.0, such as cooperation, open knowledge and management. In this sense, the evidence of cooperation (exchanged messages and coordination documents) indicates that there has been cooperation throughout the process of creating and managing knowledge.

Nevertheless, this created knowledge has been structured to facilitate its use because it enables identification of the learning activities in which it can be helpful. However, it has also been classified with a set of labels based on different views: types of learning activities, types of content, user profile and theme. This evidence constitutes support for organisational learning, specifically for the creation, organisation and distribution of knowledge.

Students have created a similar amount of knowledge for theoretical and practical activities (Table 5), and, for the most part, these resources are directed to other students who have had difficulty performing a learning activity (Table 6). Explanations, notes, examples, exercises and summaries (Table 7) are the five most utilised resources (Table 8), representing 66.6% of a total of 15 types of resources (Table 9).

The knowledge that was previously acquired individually is now created and shared with all students of the subject, which facilitates its use by students other than the group who created the knowledge. All of this contributes to increasing learning resources for the subject, which can be used in different training strategies.

The two main contributions of the Flipped Learning 4.0 model are based on the incorporation of cooperative processes to create and manage knowledge:

- This work enables validation of a new learning method for Education 4.0 based on incorporating into the FC method processes for the cooperative creation of open knowledge by the students, as well as tools to classify, organise and use it;
- The method developed is valid as a tool to develop and apply organisational learning in a subject, using the students’ experience both to improve their learning and for the continuous improvement of the subject itself.

Therefore, the Flipped Learning 4.0 model provides the university community with a method to be used in the new context of Education 4.0 and organisational learning applied to any university subject.

Future work could study the usefulness of the knowledge created for students of the same subject in another academic year or students of a different subject. The impact of this method should also be studied among students enrolled for the first time in the subject and those who were already enrolled in previous courses.
